# How do RNA molecules distinguish self from non-self?

**DOI:** 10.1073/pnas.2603593123

**Published:** 2026-04-03

**Authors:** Ofer Kimchi, Kira Mitchel, Andrew G. T. Pyo, Ned S. Wingreen, Elizabeth R. Gavis

**Affiliations:** ^a^Department of Mathematics, Courant Institute School of Mathematics, Computing, and Data Science, New York University, New York, NY 10012; ^b^Lewis-Sigler Institute for Integrative Genomics, Princeton University, Princeton, NJ 08544; ^c^Department of Molecular Biology, Princeton University, Princeton, NJ 08544; ^d^Department of Physics, Princeton University, Princeton, NJ 08544

**Keywords:** RNA, self-assembly, condensate, germ granule, phase separation

## Abstract

RNA molecules form homotypic clusters in a variety of contexts. mRNAs enriched in germ granules in *Drosophila* embryos are a canonical example, with *polar granule component* (*pgc*) mRNAs colocalized with other *pgc* mRNAs, and *nanos* mRNAs with other *nanos* mRNAs. The observation of homotypic clustering poses a conundrum: how can RNAs of a given sequence distinguish other RNAs of the same sequence from those with different sequences? Here we show in silico that RNAs can distinguish self from non-self through the presence of palindromic regions within RNA sequences, and that palindromes can mediate homotypic clustering. We further show that RNA–RNA interactions are unlikely to lead to homotypic clusters in the absence of palindromes due to a competition between intra- and intermolecular RNA structures. We explore the implications of the palindrome-based clustering hypothesis for *nanos* and *pgc* mRNAs, and suggest how it may clear up a surprising feature of *nanos* clusters. More broadly, our results indicate that the palindrome content of RNAs may be under evolutionary selection pressure across a range of contexts.

## Significance

RNA molecules frequently assemble into clusters inside cells. In organisms including fruit flies, zebrafish, and nematodes, certain RNA clusters are found to be homotypic, comprising multiple copies of the same RNA sequence. Homotypic clustering poses a puzzle, as it is by definition sequence-specific, but occurs in a wide variety of sequence contexts. We present a potential resolution, demonstrating that self-complementary stretches of RNA, termed palindromes, can mediate homotypic clustering in silico. We show that homotypically clustering sequences in *Drosophila* are enriched for strongly binding and highly accessible palindromes, and hypothesize that palindromes may be under evolutionary selection in RNAs more broadly.

Phase separation is a major driving force enabling intracellular organization. Condensates composed primarily of proteins and RNAs form and dissolve in response to cellular signals, and appear essential to biological function. While condensate research has predominantly focused on protein–protein interactions, RNAs play a role in most condensates in vivo and can form condensates in the absence of protein in vitro (1–3). Moreover, RNA-based condensates have both medical consequences and technological applications (4, 5). While many similarities exist between RNAs and proteins—both are heteropolymers with a sequence-dependent capacity for intermolecular interactions that can lead to phase separation—we wanted to explore how cluster formation may differ between RNAs and proteins.

As a model system, we specifically sought to explore homotypic RNA clusters in *Drosophila melanogaster*. Ribonucleoprotein condensates called germ granules are found in the posterior-most cytoplasm of the *Drosophila* embryo, and are crucial for inducing the formation of germ cells and for specifying germline fate (6). These granules are not homogeneous—of particular interest here is the finding that certain mRNAs within granules form homotypic clusters. Superresolution imaging has revealed that there is a high degree of spatial colocalization between certain RNAs of the same sequence (7–9). Namely, *nanos* mRNAs are more likely to be colocalized with other *nanos* mRNAs than with *pgc* mRNAs, and vice versa. These homotypic clusters range in size between 2 and several dozen RNAs (7, 9).

Despite a decade of research into this phenomenon, the mechanism behind homotypic clustering of *Drosophila* RNAs remains unknown. No individual part of the *nanos* sequence appears sufficient to mediate homotypic clustering, and homotypic cluster formation is disrupted by the addition of a variety of heterologous sequences (10). It is currently unclear whether homotypic cluster formation is driven primarily by RNA–protein or RNA–RNA interactions. Meanwhile, homotypic RNA clusters are increasingly being found in other contexts, such as in *Caenorhabditis elegans* (11, 12), in zebrafish (13, 14), and synthetically (15). Surprisingly, when *gfp* mRNA bearing the *nanos* 3′UTR was introduced into germ granules, the hybrid RNA formed homotypic clusters separate from *nanos* clusters (10).

Homotypic clusters are challenging to explain because they represent an apparent contradiction. On the one hand, homotypic cluster formation appears to be a generic phenomenon, emerging in multiple sequence contexts and independent of any particular sequence; on the other hand, these clusters are by definition sequence-specific, as the RNA sequence itself apparently contains information that directs other RNAs of the same sequence to colocalize.

Here, we present a hypothesis that resolves this apparent contradiction. We show that homotypic clustering can be explained by a feature present in RNAs but not in proteins, namely self-complementary regions of RNA, or “palindromes.” Examples of RNA palindromes include ^5′^GCAUGC^3′^ and ^5′^ACAUGU^3′^. Palindromes are common in both natural and random RNAs (*SI Appendix*). We show that palindromic regions enable generic sequence-specific RNA self-recognition through multivalent interactions, and that the sequences of *nanos* and *pgc* appear to have evolved toward palindrome-driven homotypic clustering.

**Fig. 1.**
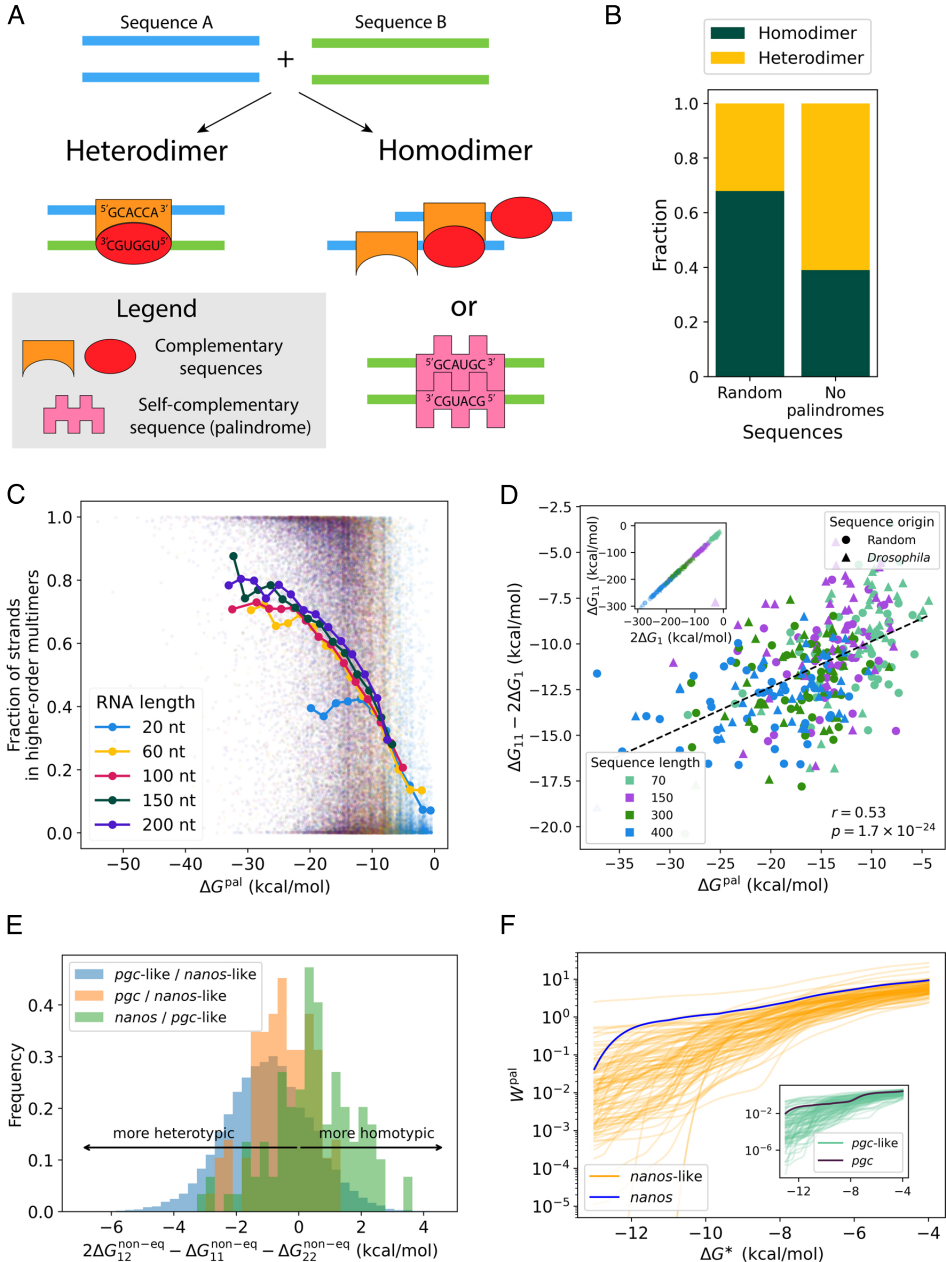
(*A*) Self-complementary regions (palindromes) facilitate homotypic interactions. (*B*) Fraction of $10^{4}$ pairs of 30 nt-long RNAs forming more homodimers or heterodimers in equilibrium, for random sequences (*Left*) and those with no palindromes ≥4 nts (*Right*). (*C*) Homomultimerization propensity of $5\times 10^{4}$ random sequences, plotted against each sequence’s palindrome binding strength. (*D*, *Inset*) Homodimer free energy ($\Delta G_{11}$) plotted against twice the monomer free energy ($2\Delta G_{1}$) for random and *Drosophila* RNAs of different lengths. (*Main*) $\Delta G_{11}-2\Delta G_{1}$ plotted against each sequence’s palindrome binding strength. (*E*) Nonequilibrium homodimerization propensities of *Drosophila* sequences length-matched to *nanos* and *pgc* are histogrammed (blue) and compared to those of *pgc* (orange) and *nanos* (green) for $\Delta G^{*}=-12$ kcal/mol. (*F*) Nonequilibrium weights of palindromes $W^{\text{pal}}$ in *nanos* (main figure) and *pgc* (*Inset*) are compared to length-matched *Drosophila* sequences as a function of $\Delta G^{*}$. Further details in *Materials and Methods*.

To illustrate our hypothesis, consider two random RNA sequences, “A” and “B.” We ask: if two molecules of A and two of B are mixed together, are they more likely to form homodimers (A bound to A and B to B) or heterodimers (A bound to B)? One might expect homodimers and heterodimers to be roughly equally likely, since a given region of A (Fig. 1*A*, orange convex shape) is roughly equally likely to be complementary to a region of B as to some other region of A (Fig. 1*A*, red oval). However, if the region selected is self-complementary (a palindrome; Fig. 1*A*, pink curve), its complement is by definition on the other copy of the same molecule. This effect leads to the striking result that short random sequences are twice as likely to form homodimers as they are to form heterodimers (Fig. 1*B*, *Left*). This result indeed relies on palindromes: considering only sequences with no palindromes, homodimers are less likely to form than heterodimers (Fig. 1*B*, *Right*).

The same principle holds beyond dimerization as well. We quantify the propensity of random sequences to form multimers of different sizes. For each sequence, we also quantify the strength of its palindromic interactions as

[1] $$\begin{array}{c} \frac{\Delta G^{\text{pal}}}{k_{\text{B}}T}=-\log(\sum_{\left\{s\right\}}e^{-\Delta G_{s}/k_{\text{B}}T}), \end{array}$$

where s represents a palindromic region of the sequence and $\Delta G_{s}$ is the free energy of two copies of the palindrome bound to one another, calculated using the nearest neighbor model (16). Across different sequence lengths, we find that the strengths of palindrome–palindrome interactions are significantly correlated ($P<10^{-300}$) with the sequence’s propensity to form higher-order homomultimers (Fig. 1*C*).

In fact, homotypic clustering based on RNA–RNA interactions is unlikely to occur at all in the absence of palindromes. To illustrate this principle, consider a naive model in which the stability of a homotypic RNA cluster of size m can be separated into the interactions of palindromic and nonpalindromic sequences: $\Delta G_{\text{m}}=\Delta G_{\text{pal}}+m\Delta G_{\text{non-pal}}$. Any nonpalindromic interaction between identical RNAs could also be present in the molecules’ monomeric states. Therefore, $\Delta G_{\text{1}}\approx \Delta G_{\text{non-pal}}$. Conversely, all base pairs of a palindrome can be satisfied only intermolecularly. The homomultimerization propensity is given by $\Delta G_{\text{m}}-m\Delta G_{\text{1}}$ (17) which is approximately equal to the strength of palindrome interactions $\Delta G_{\text{pal}}$. In other words, an RNA sequence with few palindromes has little enthalpic incentive to bind other identical molecules, as nonpalindromic intermolecular bonds can largely be satisfied just as readily by intramolecular bonds.

To validate this conceptual understanding, we consider both random RNA sequences and size-matched *Drosophila* RNAs, and compare their monomer and homodimer equilibrium free energies. We find that, in agreement with the above conceptual analysis, homodimer free energies are highly correlated to the monomer free energies (Fig. 1*D*, *Inset*), and that the difference between the two (i.e., $G_{\text{11}}-2\Delta G_{\text{1}}$) is correlated to the strength of palindrome–palindrome interactions (Fig. 1*D*) with r=0.53 and $P=10^{-24}$ (computed across all lengths and sequence origins). In other words, the properties of palindromic regions determine the propensity of RNAs to form homotypic dimers.

So far, we have demonstrated that in equilibrium, palindromes predict RNA homomultimerization. However, to understand in vivo homotypic clustering, we also need to explore out-of-equilibrium effects, as prior work has shown *nanos* RNAs cluster without extensive changes to their structures (18). The out-of-equilibrium nature of RNA hybridization is poorly understood (19). Nevertheless, we can make progress by considering the strength of an initial binding event between two folded RNAs, hypothesizing that intermolecular interactions which do not require intramolecular structure unfolding will dominate out-of-equilibrium binding. We define the strength of such an initial binding event as a function of complementary regions i and j on the two molecules:

[2] $$\begin{array}{c} \frac{\Delta G^{\text{non-eq}}}{k_{\text{B}}T}=-\log(\sum_{\left\{i,j\right\}}p_{i}^{\text{free}}p_{j}^{\text{free}}e^{-e^{\left(\Delta G_{\mathrm{ij}}-\Delta G^{*}\right)/k_{\text{B}}T}}), \end{array}$$

where $p_{i}^{\text{free}}$ is the probability that region i is accessible in equilibrium and $\Delta G_{\mathrm{ij}}$ is the binding strength of the two regions. The double exponential describes the probability that the bond does not dissociate within a time $t^{*}=\exp\left(-\Delta G^{*}/k_{\text{B}}T\right)/k$ with $k\approx 10^{7}$ s^−1^ an “attempt frequency” (20). This form arises because waiting times follow an exponential distribution in $Z=\;\exp(-\Delta G_{\mathrm{ij}}/k_{\text{B}}T)$.

We estimate $\Delta G_{\mathrm{ij}}$ using the nearest neighbor model (16) and $p^{\text{free}}$ as the probability each region is unbound in the equilibrium secondary structure, found by sampling $10^{5}$ structures from the equilibrium distribution of each molecule calculated using NUPACK (21). To uncover the features that lead to *nanos* and *pgc* homomultimers, we compare these sequences to ∼100*Drosophila* mRNAs of similar lengths to *nanos* and *pgc* (*Materials and Methods*).

We find that out of equilibrium, heterodimerization is typically favored compared to homodimerization (Fig. 1*E*). Specifically, we measure the nonequilibrium binding between each *nanos*-like sequence (i.e., length-matched to *nanos*) and each *pgc*-like sequence, to calculate $\Delta G_{12}^{\text{non-eq}}$. This value is compared to the sequences’ self-binding ($\Delta G_{11}^{\text{non-eq}}+\Delta G_{22}^{\text{non-eq}}$). Strikingly, the homodimerization propensities of both *nanos* (green) and *pgc* (orange) are higher than those of typical *Drosophila* sequences (blue) (Fig. 1*E*).

Can we understand the relative propensities of *nanos* and *pgc* to form homotypic interactions as resulting from their palindromes? We consider the nonequilibrium weight of palindromic regions, defined as $W^{\text{pal}}\equiv \;\exp\left(-\Delta G^{\text{non-eq; pal}}/k_{\text{B}}T\right)$, where $\Delta G^{\text{non-eq; pal}}$ is given by Eq. **2** with the sum constrained to only the self-binding of palindromic regions. In Fig. 1*F*, we plot this weight for *nanos* (*Main figure*) and *pgc* (*Inset*) as a function of $\Delta G^{*}$, and compare these to the length-matched *Drosophila* sequences considered previously. We find that across a range of values of $\Delta G^{*}$, *nanos* and *pgc* have a higher weight of palindromic regions than other sequences of similar lengths.

The multivalency of palindromes has experimental implications. We find that both *nanos* and *pgc* have more strongly binding ($\Delta G\leq \Delta G^{*}$) and highly accessible ($p^{\text{free}}\geq 10^{-1}$) palindromic regions than 95% of the other length-matched *Drosophila* sequences, across a wide range of $\Delta G^{*}$ values, particularly (for *nanos*) between −12.5 kcal/mol $\leq \Delta G^{*}\leq -8.5$ kcal/mol. Notably, for $\Delta G^{*}\geq -12.5$ kcal/mol, *nanos* has multiple accessible and strongly binding palindromes. This implies that deleting a single region may not affect its clustering properties, as has been seen experimentally (10).

We note that the direct application of Eq. **2** to arbitrary sequences is limited by the inaccuracy of current in silico tools when predicting the structures of long RNAs in vivo (22). Applying Eq. **2** to *nanos* and *pgc* sequences in *Drosophila virilis* and *Drosophila pseudoobscura*, we find that only *pgc* in those species is enriched for strongly binding and accessible palindromes, and displays a tendency toward homomultimerization (*SI Appendix*, Fig. S1 *A*–*D*). We attribute the discrepancy to the inaccuracy of RNA structure prediction, which directly affects $p^{\text{free}}$. Nevertheless, we find here as well a close connection between homotypic clustering propensity and palindrome content (*SI Appendix*, Fig. S1 *A*–*D*).

Similarly, applying Eq. **2** to a broader set of sequence pairs reported to homotypically cluster in vivo yields a range of predicted homodimerization propensities (*SI Appendix*, Fig. S1*E*). However, taken together, these sequences still exhibit a significantly higher predicted homodimerization propensity than the null model of palindrome-free sequences ($P=1\times 10^{-5}$ with one-sided Student’s *t* test).

In summary, we have shown in silico that the presence of palindromes can enable RNAs to distinguish copies of themselves from other RNA sequences. Both in and out of equilibrium, the homotypic binding propensities of RNAs can be understood as arising to a large extent from palindromic regions of the sequences. Our results can explain how even RNA sequences without specialized properties can exhibit homotypic clustering, as has been seen experimentally with clustering of *gfp* mRNA in *Drosophila* (10). Furthermore, we have hypothesized that palindromes can be evolutionarily selected for, and that *Drosophila* mRNAs in germ granules use this strategy to achieve robust homotypic clustering of *nanos* and *pgc*. This framework suggests a potential explanation for the surprising finding that no particular sequence drives homotypic clustering in *Drosophila* embryogenesis (10), as *nanos* contains many potential palindrome–palindrome interactions throughout its UTRs and coding region.

Many questions remain to be explored. Most importantly, we lack a quantitative predictive model for palindrome-induced phase separation. How much palindromic content is necessary to induce homomultimerization? So far, such a framework has only been developed for sequences with a single type of palindrome (23). Our findings indicate that a quantitative model for RNA homotypic clustering need not necessarily consider the entirety of the sequence, but can be effectively captured by focusing on palindromic regions.

Of course, our results do not negate the possibility of homotypic clustering behavior driven by nonpalindromic RNA–RNA interactions, and future research into RNA–protein interactions mediating homotypic clustering is warranted. Experiments to test the framework we present would include measuring the homotypic clustering properties of synthetic sequences designed to have a minimal number of palindromes, as well as those designed to have strongly binding palindromic regions unoccluded by secondary structure. Our framework predicts that the former sequences would not exhibit homotypic clustering, and the latter would strongly cluster.

Our work demonstrates that palindromes enable a generic method of RNA self-recognition. We therefore expect palindromes to be under strong selection pressure—either positive or negative—across a broad range of RNA contexts.

## Materials and Methods

NUPACK was used to calculate free energies, multimerization propensities, and equilibrium accessibilities (21). Code is available at https://github.com/ofer-kimchi/homotypic (24).

### Sequences Used.

To compare *nanos* and *pgc* to similar sequences, we first downloaded *Drosophila melanogaster* mRNA sequences from the NCBI RefSeq assembly GCF_000001215.4. We sorted these sequences by length, and chose ∼100 sequences of lengths similar to those of *nanos* and *pgc*. Specifically, we found 104 sequences of length L such that $L_{\mathrm{pgc}}-5<L<L_{\mathrm{pgc}}+5$, and 106 sequences of length L such that $L_{\mathrm{nanos}}-8<L<L_{\mathrm{nanos}}+8$. We then used the Smith-Waterman algorithm (with match, mismatch, and gap score contributions of +1,−2,−2, respectively) to compare the sequence similarities of these sequences. We found that 6 of the sequences length-matched to *nanos* and 2 of the sequences length-matched to *pgc* sequences were near-duplicates of another sequence in our list. (We note that it was straightforward in this case to distinguish between pairs of near-duplicate sequences, which had sequence similarity scores close to their lengths, from pairs of dissimilar sequences, which had scores ≲20.) We removed these duplicates in our analyses, remaining with 100 sequences of lengths (approximately) equal to that of *nanos*, and 102 sequences for *pgc*.

### Fig. 1*B* Simulation Details.

Random RNAs were generated with each nucleotide at each position being chosen independently with a 25% probability of A, C, G, or U. Given two such random RNAs, NUPACK was used to calculate the total free energy of homodimer formation ($\Delta G_{\text{hom}}=\Delta G_{11}+\Delta G_{22}$) and the total free energy of heterodimer formation ($\Delta G_{\text{het}}=2\Delta G_{12}$). The plot shows the fraction of sequence pairs for which $\Delta G_{\text{hom}}<\Delta G_{\text{het}}$ (green; *Bottom*) or $\Delta G_{\text{hom}}>\Delta G_{\text{het}}$ (yellow; *Top*). To generate palindrome-free sequences, we follow the same procedure, but repeatedly generate a new sequence if the random RNA contains any palindromes of length ≥4.

### Fig. 1*C* Simulation Details.

Random RNAs of different lengths were generated, with $10^{4}$ sequences of each length. NUPACK was used to calculate the partition function of homomultimers of each sequence, up to a maximum cluster size of 10. This cluster size was chosen as considering larger clusters becomes increasingly expensive in storage space and calculation time, and we found minimal changes in our results when considering smaller maximum cluster sizes. These partition functions were used along with an input concentration to calculate the fraction of strands in clusters of each size (17). Since we are interested in the relationship between the fraction of strands in higher-order multimers and $\Delta G^{\text{pal}}$, the absolute value of this concentration is unimportant, as long as the same concentration is used for sequences of the same length. To control for the length dependence of clustering, the concentration of each sequence of length L was set to $4\times {\left(20/L\right)}^{2.5}$ mM. Changes to this concen- tration lead to the same qualitative results. We defined “higher-order” multimers as clusters of at least four molecules; our results are robust to changes in this threshold. Solid curves represent the average multimerization propensity calculated for each sequence length in a bin of width 2 kcal/mol, neglecting bins with fewer than 30 datapoints.

### Fig. 1*D* Simulation Details.

Plots show sequences of four different lengths (20 sequences of each length) either randomly generated (circles) or naturally occurring *Drosophila* RNAs (triangles). The lengths of the *Drosophila* RNAs are within 6 nts of the length specified. Each sequence’s palindrome binding strength was calculated using Eq. **1** and the nearest-neighbor model.

### Fig. 1*E* Simulation Details.

We constructed the ∼100 × 100 matrix of nonequilibrium heterodimer binding strengths between sequences length-matched to *nanos* and *pgc* (we will refer to these as *nanos*-like and *pgc*-like here). For each *nanos*-like/*pgc*-like pair, we calculated $2\Delta G_{12}^{\text{non-eq}}-$$\left(\Delta G_{11}^{\text{non-eq}}+\Delta G_{22}^{\text{non-eq}}\right)$, meaning the non-equilibrium propensity of the sequence pair to form heterodimers as opposed to homodimers. This quantity is histogrammed in blue. In orange, we histogram the propensity of *pgc* to form heterodimers as opposed to homodimers when presented with each *nanos*-like sequence (calculated in the same manner), and in green, we histogram the propensity of *nanos* to form heterodimers as opposed to homodimers when presented with each *pgc*-like sequence. our results are largely insensitive to the choice of $\Delta G^{*}=-12$ kcal/mol used, with panel *F* giving an intuition as to why this is the case.

Further methodology details may be found in *SI Appendix*.

## Supplementary Material

Appendix 01 (PDF)

## Data Availability

Code data have deposited in GitHub (https://github.com/ofer-kimchi/homotypic) (24). All other data are included in the manuscript and/or supporting information.
