# Supplementary material for: How do RNA molecules distinguish self from non-self?
Source: Proc Natl Acad Sci U S A. 2026 Apr 3;123(15):e2603593123. doi: 10.1073/pnas.2603593123 (PMC13079385; doi:10.1073/pnas.2603593123)
Supplement: Supplementary file 1 — Appendix 01 (PDF) [file pnas.2603593123.sapp.pdf]

Supporting Information for

How do RNA molecules distinguish self from non-self?

Ofer Kimchi, Kira Mitchel, Andrew G. T. Pyo, Ned S. Wingreen, Elizabeth R. Gavis

Ofer Kimchi.
E-mail: ok2294@nyu.edu

This PDF file includes:

Supporting text
Fig. S1

Supporting Information Text

1. Defining a region for binding

In our definitions of $\Delta G^{\text{non-eq}}$ (Eq. 2) and of $\Delta G^{\text{non-eq; pal}}$, we sum over groups of consecutive nts, which we call "regions". (In the former case, we sum over over pairs of complementary regions on two molecules; in the latter, over individual palindromic regions on a single molecule.) In this note, we discuss a nuance to this sum which may not be apparent. In aid of clarity, we present this nuance with respect to the calculation of $\Delta G^{\text{non-eq; pal}}$, but the same principle applies (and was implemented) for the calculation of $\Delta G^{\text{non-eq}}$.

Consider, as an example, a palindromic region of length $L > 2$. It is apparent that by removing a nucleotide on each end of this region, we can arrive at a second palindromic region of length $L - 2$ that is entirely contained within the first. As $\Delta G^{\text{non-eq}}$ is meant to consider the strength of an initial binding event between two RNA molecules, it is unreasonable to include both of these regions in the sum. This is because interactions between two RNA molecules initiate at a particular nt on each strand and extend from there. For this reason, considering both of these palindromic regions independently would overcount interactions that initiated at one of the nt pairs common to both.

A naive resolution would be to consider the longest palindrome and ignore its component palindromes, as the longest palindrome would have the strongest binding strength. However, this is not a valid resolution in the context of our non-equilibrium model. To illustrate this, consider a long palindrome with a binding strength $\Delta G < \Delta G^*$ but with the two nts on its ends typically bound up in secondary structure such that the probability that the full palindrome is accessible in the folded state of the molecule, p^{free}, is close to 0. For such a palindrome, its contribution to $\Delta G^{\text{non-eq}}$, which is proportional to $(p^{\text{free}})^2$, would be negligible. However, this doesn't mean that this palindrome is out of play! In particular, it may well be the case that there is a shorter palindrome contained within our initial region of interest with a non-negligible value of p^{free}, and which nevertheless has a binding strength $< \Delta G^*$.

For this reason, in our calculations of $\Delta G^{\text{non-eq}}$ and $\Delta G^{\text{non-eq; pal}}$, we group nested regions together, and for each group, consider only the single "subregion" with the maximum contribution.

We note that the argument made above does not apply to Eq. 1, which can be calculated while considering all palindromic regions independently, including those nested within one another.

2. The prevalence of palindromes

Our main finding is that when it comes to homotypic clustering driven by RNA-RNA interactions, the entire RNA sequence is not equally important. To understand homotypic clustering, one can mostly focus on a small subset of that sequence: namely, palindromes. In this section we calculate how much smaller this subset is. In other words, how many palindromes of length $2l$ nts should we expect for a sequence comprising L nts?

To illustrate the answer, we will consider $l = 3$. To generate a palindrome of length 6 nts, there are no constraints on the first 3 nts, but the following 3 nts must each be complementary to their respective partner. Including G-U pairing (as we do here), the probability that a given pair of nts will be complementary is 3/8. Thus, there is a probability of $(3/8)^3$ that a given set of 6 nts will be palindromic. For the palindrome to be in total 6 nts long, the next nt must not extend the palindrome, leading to a factor of (5/8). Thus, the number of palindromes of length 6 nts in a sequence of length L is approximately $\left(\frac{3}{8}\right)^3 \left(\frac{5}{8}\right) L$.

A more precise estimate is achieved by recognizing that the first three nts of a palindrome cannot simultaneously be part of the first three nts of a second palindrome. Thus, the expected number of palindromes of length 6 in a sequence of length L, fL, must satisfy the relation

$$fL = \left(\frac{3}{8}\right)^3 \left(\frac{5}{8}\right) (L - 3fL), \qquad [1]$$

leading to $f = \frac{135}{4501} \approx 0.03$. This value is confirmed by simulating random sequences. The standard deviation of the number of palindromes of length 6 in a sequence of length L is approximately $\sqrt{fL} \approx 0.17\sqrt{L}$.

More generally, given an average probability p of complementarity between two nts, the number of palindromes of length $2l$ in a sequence of length L is given by fL with

$$f = \left[l + \frac{1}{(1-p)p^l}\right]^{-1}. \qquad [2]$$

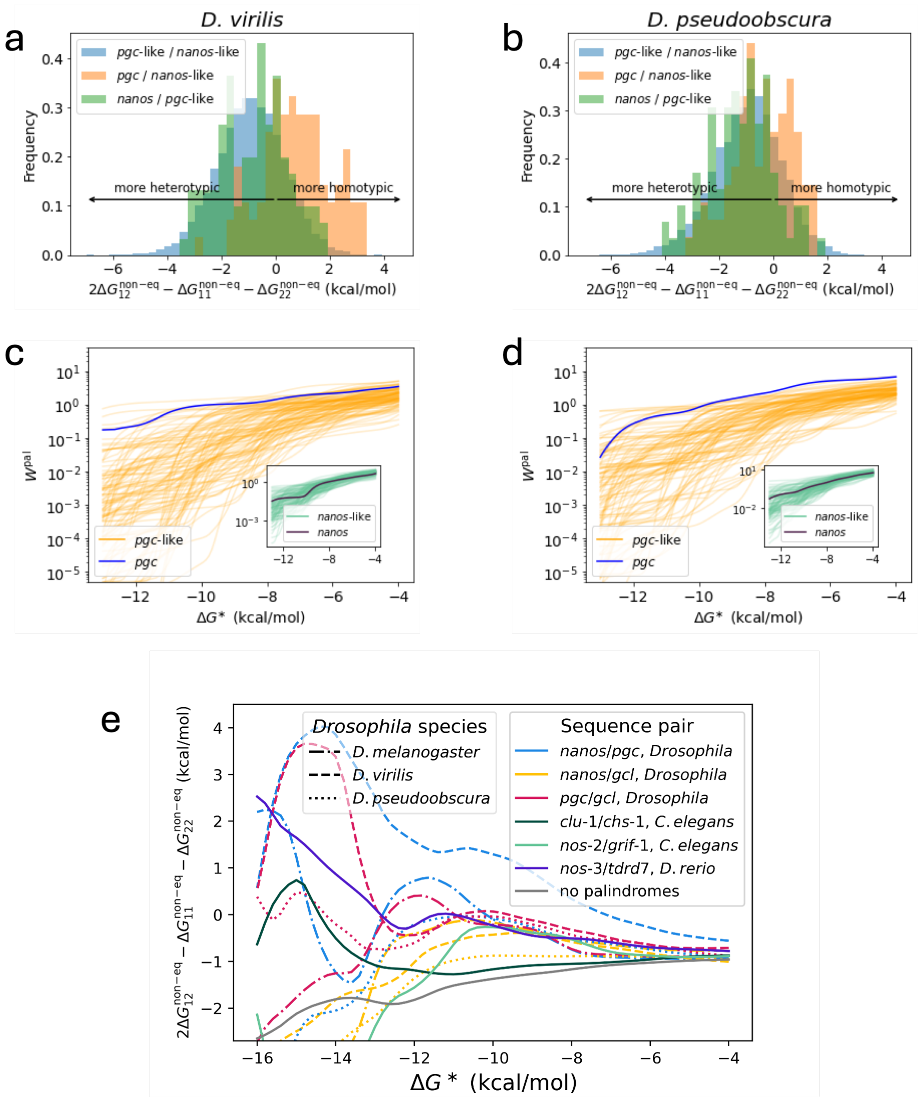

Fig. S1. a-d. The same methodology applied to Figs. 1e,f is applied to compare *nanos* and *pgc* with 100 other RNAs of similar lengths in *Drosophila virilis* (left) and *pseudoobscura* (right). **a, b**: histograms of the non-equilibrium propensities of RNAs to form homodimers (positive numbers) vs. heterodimers (negative numbers). Blue: all possible pairwise interactions; orange: propensities of *pgc* to form homodimers vs heterodimers with RNAs of same length as *nanos*; green: propensity of *nanos* to form homodimers vs heterodimers of same length as *pgc*. **c, d**: The non-equilibrium weights of palindromic regions in *pgc* (main figure) and *nanos* (inset) are compared to those of *D. virilis* (c) and *D. pseudoobscura* (d) of the same lengths, for different values of the parameter ΔG^*. **e.** We use Eq. 2 to calculate the non-equilibrium binding strengths of different pairs of sequences, and plot the propensities of the RNAs to form homodimers (positive numbers) vs. heterodimers (negative numbers) as a function of ΔG^*. We show the results for different sequence pairs: *nanos*/*pgc* (blue), *nanos*/*gcl* (yellow), and *pgc*/*gcl* (red) pairs from *D. melanogaster* (dash-dotted), *D. virilis* (dashed), and *D. pseudoobscura* (dotted), as well as the *clu*-1/*chs*-1 pair (solid; dark green) and the *nos*-2/*grif*-1 pair (solid; light green), both from *C. elegans*, and the *nos*-3/*tdrd7* pair from *D. rerio* (solid; purple). In addition, we plot as a negative control the mean of 100 pairs of sequences of length 1000 nts designed to have no palindromes of length > 4 nts (gray solid line). These palindrome-free sequences were generated by starting with a random sequence and successively shuffling regions containing palindromes. The difference between the natural and palindrome-free sequences at $\Delta G^* = -12$ kcal/mol is statistically significant with $p = 10^{-5}$, calculated using the one-sided Student's *t*-test.
